# The land snails (Mollusca, Gastropoda) of Kea island (Aegean, Greece)

**DOI:** 10.3897/BDJ.10.e87720

**Published:** 2022-09-09

**Authors:** Leonidas Maroulis, Katerina Vardinoyannis, Danae Karakasi, Konstantinos Proios, Moissis Mylonas, Kostas A Triantis

**Affiliations:** 1 University of Crete, Heraklion, Greece University of Crete Heraklion Greece; 2 Natural History Museum of Crete, Heraklion, Greece Natural History Museum of Crete Heraklion Greece; 3 Faculty of Biology, Department of Ecology and Systematics, National and Kapodistrian University of Athens, Athens, Greece Faculty of Biology, Department of Ecology and Systematics, National and Kapodistrian University of Athens Athens Greece

**Keywords:** Aegean archipelago, biodiversity, land molluscs, taxonomy

## Abstract

**Background:**

Kea is the westernmost island of the Cyclades and is located between Syros and Attica, in central Greece. In this work, we have resampled the island after 43 years – i.e. when the island was first fully sampled – and we present its complete land snail fauna.

**New information:**

We report 42 land snail species with 10 species being new records for the island. Based on our results we draw attention to the fact that sampling for land snails should be done during the wet period in order to survey the complete malacofauna in an island or a region. For such a complete survey, collection and inspection of soil and litter are also necessary. Finally, increased sampling effort through regular resurveys is a necessary prerequisite in order to effectively assess the temporal dynamics of biodiversity patterns.

## Introduction

Land molluscs and slugs (henceforth land snails) comprise an important component of global biodiversity, representing one of the most species-rich groups of terrestrial animals with approximately 28,000 recognised species (MolluscaBase 2022). Although poor dispersers (Cameron 2013, Cameron 2016), land snails are found almost everywhere on Earth (Cameron 2016) and occupy a wide range of habitat types such as deserts, forests, shrublands and alpine meadows.

For their area, islands host a disproportionate high number of species of many taxa (Fernández-Palacios et al. 2021, including land snails (Cowie 2004, Cameron et al. 2013). Global estimates of insular land snail richness, based on faunas of 727 islands, exceed 11,000 species – that is approximately 48% of all known land snail species – on less than 3% of the globe’s land mass, with 75% of them being single island endemics (Proios et al. 2021). Similarly, the Aegean Sea islands hold a unique malacofauna of 419 species, with 51% being regional endemics (Vardinoyannis and Mylonas 2019). Consequently, the Aegean Islands have long attracted scientific attention, having served as a model system for understanding the biogeography of the taxon (Fuchs and Käufel 1936, Heller 1976, Mylonas 1982, Vardinoyannis 1994, Botsaris 1996, Welter-Schultes and Williams 1999, Hausdorf and Hennig 2005, Triantis et al. 2005, Triantis et al. 2008, Goudeli et al. 2021). The systematic and intense study of land snails of the Greek islands, especially during the last four decades, has resulted in more than 150 islands being considered as well-sampled.

However, most of the island faunas have been studied through one or a limited number of field trips and thus our knowledge about their temporal dynamics is limited. In this framework, we studied and herein present the terrestrial malacofauna of Kea (Cyclades, Aegean Sea, Greece), an island that had been previously fully sampled in 1979.

## Materials and methods

### Study Area

Kea belongs to the Cyclades island group and is located 19.5 km off the mainland of Attica (Map 1). It is 19 km long (from north to south) and 9 km wide (from west to east), overall possessing an area of 131.7 km², with its highest elevation being 560 m. Kea is, geologically, part of the Attic-Cycladic Unit (Papanikolaou 2021) and consists almost exclusively of metamorphic rocks, mainly schist and marble remains (Yoxas et al. 2011). The island’s morphology is characterised by its intense topographic relief and the main (snail) habitat types are phrygana, maquis and *Quercusithaburensis* forests.

### Sampling

**Figure 1.**
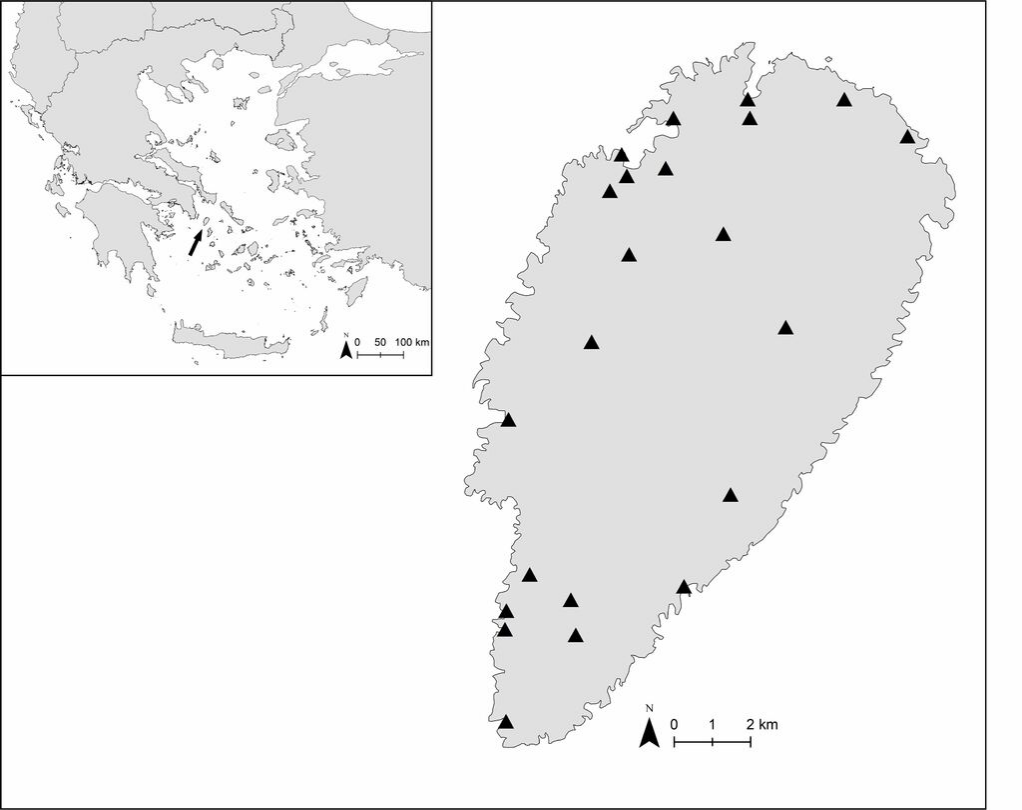
Map of Kea, the island’s location in the Aegean archipelago (Greece) and the 22 localities sampled for land snails shown with a triangle.

Sampling of land snails was carried out between 4 and 8 November 2021 by five malacologists (LM, KV, KP, MM and KAT) at 22 localities (Fig. 1, Suppl. material 1). We collected samples from a great spectrum of ecosystems, including shrublands (phrygana, maquis or mixed), oak forests, riparian vegetation around streams, cultivations, urban and peri-urban areas. Moreover, we gathered soil and litter under various plants. The collected live specimens were drowned in water and then preserved in 75% ethanol. Some of the individuals were also preserved in 96% ethanol for future molecular analyses. In the laboratory, the collected litter and soil were left to dry and then sieved. A 5-level test sieve was used (with mesh sizes of 5, 2.5, 1.7, 1 and 0.5 mm). Material passing through the 0.5 mm mesh was discarded, while the material obtained from each sieve was examined for small snails under a magnifying lens and good lighting. Shell morphology and reproductive systems were studied to perform identification at the species-level. The entire material collected is deposited in the malacological collection of the Natural History Museum of Crete (NHMC). Additionally, in order to supplement our recovered species list with species potentially not found, but reported in previous works, we re-examined material stored in the malacological collection of NHMC. Previous works reporting snails from the island (Fuchs and Käufel 1934, Fuchs and Käufel 1936, Mylonas 1982, Riedel 1992, Wiktor 2001) were also consulted for distributional data.

## Checklists

### Land snail species from Kea island

#### Albinaria discolor

(Pfeiffer, 1846)

00C05170-EDBC-599A-8E2D-5534F4A843BE

##### Materials

- **Type status:** Other material. **Taxon:** scientificName: *Albinariadiscolor* (Pfeiffer, 1846); order: Stylommatophora; family: Clausiliidae; **Location:** continent: Europe; islandGroup: Cyclades; island: Kea; country: Greece; countryCode: GR; **Identification:** identifiedBy: M Mylonas; **Event:** eventDate: 4/11-8/11/2021; **Record Level:** institutionCode: NHMC

#### Albinaria turrita

(Pfeiffer, 1850)

F5F2E24C-8C2D-5891-99C2-380DFD16C774

##### Materials

- **Type status:** Other material. **Taxon:** scientificName: *Albinariaturrita* (Pfeiffer, 1850); order: Stylommatophora; family: Clausiliidae; **Location:** continent: Europe; islandGroup: Cyclades; island: Kea; country: Greece; countryCode: GR; **Identification:** identifiedBy: M Mylonas; **Event:** year: 1979; **Record Level:** institutionCode: NHMC

#### Cantareus apertus

(Born, 1778)

572CEBF2-DC59-53B7-AB0E-5397FCEF2567

##### Materials

- **Type status:** Other material. **Taxon:** scientificName: *Cantareusapertus* (Born, 1778); order: Stylommatophora; family: Helicidae; **Location:** continent: Europe; islandGroup: Cyclades; island: Kea; country: Greece; countryCode: GR; **Identification:** identifiedBy: L Maroulis; **Event:** eventDate: 4/11-8/11/2021; **Record Level:** institutionCode: NHMC

#### Caracollina lenticula

(Michaud, 1831)

2C24819D-70DE-5AE7-A07E-A9062AD30671

##### Materials

- **Type status:** Other material. **Taxon:** scientificName: *Caracollinalenticula* (Michaud, 1831); order: Stylommatophora; family: Trissexodontidae; **Location:** continent: Europe; islandGroup: Cyclades; island: Kea; country: Greece; countryCode: GR; **Identification:** identifiedBy: D Karakasi; **Event:** eventDate: 4/11-8/11/2021; **Record Level:** institutionCode: NHMC

#### Candidula syrensis

(Pfeiffer, 1846)

C4E7BC7A-16DE-500B-A08D-BC1A89E8C27C

##### Materials

- **Type status:** Other material. **Taxon:** scientificName: *Candidulasyrensis* (Pfeiffer, 1846); order: Stylommatophora; family: Geomitridae; **Location:** continent: Europe; islandGroup: Cyclades; island: Kea; country: Greece; countryCode: GR; **Identification:** identifiedBy: M Mylonas; **Event:** eventDate: 4/11-8/11/2021; **Record Level:** institutionCode: NHMC

#### Cecilioides acicula

(Müller, 1774)

61F12639-CD29-5492-8E0E-74E195E598F7

##### Materials

- **Type status:** Other material. **Taxon:** scientificName: *Cecilioidesacicula* (Müller, 1774); order: Stylommatophora; family: Ferussaciidae; **Location:** continent: Europe; islandGroup: Cyclades; island: Kea; country: Greece; countryCode: GR; **Identification:** identifiedBy: M Mylonas; **Event:** eventDate: 4/11-8/11/2021; **Record Level:** institutionCode: NHMC

#### Cecilioides tumulorum

(Bourguignat, 1856)

020B61E4-BD28-575C-96FE-1AEB2DC7E277

##### Materials

- **Type status:** Other material. **Taxon:** scientificName: *Cecilioidestumulorum* (Bourguignat, 1856); order: Stylommatophora; family: Ferussaciidae; **Location:** continent: Europe; islandGroup: Cyclades; island: Kea; country: Greece; countryCode: GR; **Identification:** identifiedBy: M Mylonas; **Event:** eventDate: 4/11-8/11/2021; **Record Level:** institutionCode: NHMC

##### Notes

New record from Kea.

#### Cernuella virgata

(Da Costa, 1778)

4D661F76-B91C-593B-A0CC-938F1122F156

##### Materials

- **Type status:** Other material. **Taxon:** scientificName: *Cernuellavirgata* (Da Costa, 1778); order: Stylommatophora; family: Geomitridae; **Location:** continent: Europe; islandGroup: Cyclades; island: Kea; country: Greece; countryCode: GR; **Identification:** identifiedBy: M Mylonas; **Event:** eventDate: 4/11-8/11/2021; **Record Level:** institutionCode: NHMC

##### Notes

*Xerocrassacretica* reported by Martens 1889 and Fuchs and Käufel 1934 is a misidentification of *Cernuellavirgata*, which is very common on the island and some of its populations share shell characteristics with *Xerocrassacretica*.

#### Chondrula bergeri

(Roth, 1839)

BC1D9AC3-8266-58F6-BC5B-AE05FD3B63C3

##### Materials

- **Type status:** Other material. **Taxon:** scientificName: *Chondrulabergeri* (Roth, 1839); order: Stylommatophora; family: Enidae; **Location:** continent: Europe; islandGroup: Cyclades; island: Kea; country: Greece; countryCode: GR; **Identification:** identifiedBy: D Karakasi; **Event:** eventDate: 4/11-8/11/2021; **Record Level:** institutionCode: NHMC

#### Chondrus zebrulus

(Férussac, 1821)

EEBD580A-C89D-55C8-BF33-3DE79BF09005

##### Materials

- **Type status:** Other material. **Taxon:** scientificName: *Chondruszebrulus* (Férussac, 1821); order: Stylommatophora; family: Enidae; **Location:** continent: Europe; islandGroup: Cyclades; island: Kea; country: Greece; countryCode: GR; **Identification:** identifiedBy: M Mylonas; **Event:** eventDate: 4/11-8/11/2021; **Record Level:** institutionCode: NHMC

#### Cochlicella acuta

(Müller, 1774)

E16BF6A3-9B6F-5F68-8B05-DB88D9DE9BDC

##### Materials

- **Type status:** Other material. **Taxon:** scientificName: *Cochlicellaacuta* (Müller, 1774); order: Stylommatophora; family: Geomitridae; **Location:** continent: Europe; islandGroup: Cyclades; island: Kea; country: Greece; countryCode: GR; **Identification:** identifiedBy: L Maroulis; **Event:** eventDate: 4/11-8/11/2021; **Record Level:** institutionCode: NHMC

#### Cornu aspersum

(Müller, 1774)

8D9849E7-C0B1-58B4-AF2F-E55619108323

##### Materials

- **Type status:** Other material. **Taxon:** scientificName: *Cornuaspersum* (Müller, 1774); order: Stylommatophora; family: Helicidae; **Location:** continent: Europe; islandGroup: Cyclades; island: Kea; country: Greece; countryCode: GR; **Identification:** identifiedBy: L Maroulis; **Event:** eventDate: 4/11-8/11/2021; **Record Level:** institutionCode: NHMC

#### Deroceras keanense

van Regteren Altena, 1973

1DCE4AB1-E202-50C2-8E17-C6A2A0EF9DD2

##### Materials

- **Type status:** Other material. **Taxon:** scientificName: *Deroceraskeanense* van Regteren Altena, 1973; order: Stylommatophora; family: Agriolimacidae; **Location:** continent: Europe; islandGroup: Cyclades; island: Kea; country: Greece; countryCode: GR; **Identification:** identifiedBy: K Vardinoyannis; **Event:** eventDate: 4/11-8/11/2021; **Record Level:** institutionCode: NHMC

##### Notes

*Derocerasberythense* is not distributed in Greece according to Wiktor (2001) and all the records of *D.berythense* from Kea should be regarded as belonging to *D.keanense*.

#### Deroceras laeve

(Müller, 1774)

63DFD2D0-5FFE-5672-AC89-2B30955DF2EC

##### Materials

- **Type status:** Other material. **Taxon:** scientificName: *Deroceraslaeve* (Müller, 1774); order: Stylommatophora; family: Agriolimacidae; **Location:** continent: Europe; islandGroup: Cyclades; island: Kea; country: Greece; countryCode: GR; **Identification:** identifiedBy: A Wiktor; **Event:** year: 1979

#### Deroceras pseudopanormitanum

Wiktor, 1984

521DA7F0-B48A-59B1-A563-888C306CAF22

##### Materials

- **Type status:** Other material. **Taxon:** scientificName: *Deroceraspseudopanormitanum* (Wiktor, 1984); order: Stylommatophora; family: Agriolimacidae; **Location:** continent: Europe; islandGroup: Cyclades; island: Kea; country: Greece; countryCode: GR; **Identification:** identifiedBy: K Vardinoyannis; **Event:** eventDate: 4/11-8/11/202; **Record Level:** institutionCode: NHMC

##### Notes

New record from Kea and the Aegean Islands.

#### Deroceras seriphium

Wiktor & Mylonas, 1981

9981FB19-739F-5FFB-8D43-424504CD69FB

##### Materials

- **Type status:** Other material. **Taxon:** scientificName: *Derocerasseriphium* Wiktor & Mylonas, 1981; order: Stylommatophora; family: Agriolimacidae; **Location:** continent: Europe; islandGroup: Cyclades; island: Kea; country: Greece; countryCode: GR; **Identification:** identifiedBy: K Vardinoyannis; **Event:** eventDate: 4/11-8/11/2021; **Record Level:** institutionCode: NHMC

#### Eobania vermiculata

(Müller, 1774)

21B17067-85BB-5456-A21B-EA244DF24B07

##### Materials

- **Type status:** Other material. **Taxon:** scientificName: *Eobaniavermiculata* (Müller, 1774); order: Stylommatophora; family: Helicidae; **Location:** continent: Europe; islandGroup: Cyclades; island: Kea; country: Greece; countryCode: GR; **Identification:** identifiedBy: L Maroulis; **Event:** eventDate: 4/11-8/11/2021; **Record Level:** institutionCode: NHMC

#### Granopupa granum

(Draparnaud, 1801)

BE9C021A-E136-525C-A79F-69FC158A7A0E

##### Materials

- **Type status:** Other material. **Taxon:** scientificName: *Granopupagranum* (Draparnaud, 1801); order: Stylommatophora; family: Chondrinidae; **Location:** continent: Europe; islandGroup: Cyclades; island: Kea; country: Greece; countryCode: GR; **Identification:** identifiedBy: D Karakasi; **Event:** eventDate: 4/11-8/11/2021; **Record Level:** institutionCode: NHMC

#### Helix figulina

Rossmässler, 1839

D7325785-20B0-5161-AE57-93ED9EE9D663

##### Materials

- **Type status:** Other material. **Taxon:** scientificName: *Helixfigulina* Rossmässler, 1839; order: Stylommatophora; family: Helicidae; **Location:** continent: Europe; islandGroup: Cyclades; island: Kea; country: Greece; countryCode: GR; **Identification:** identifiedBy: L Maroulis; **Event:** eventDate: 4/11-8/11/2021; **Record Level:** institutionCode: NHMC

#### Idyla bicristata

(Rossmässler, 1839)

0870F528-CCB9-5D22-AC61-B3C2DE94BCCC

##### Materials

- **Type status:** Other material. **Taxon:** scientificName: *Idylabicristata* (Rossmässler, 1839); order: Stylommatophora; family: Clausiliidae; **Location:** continent: Europe; islandGroup: Cyclades; island: Kea; country: Greece; countryCode: GR; **Identification:** identifiedBy: D Karakasi; **Event:** eventDate: 4/11-8/11/2021; **Record Level:** institutionCode: NHMC

#### Lauria cylindracea

(Da Costa, 1778)

CDA415E2-6892-579D-AC67-AEC3117C3652

##### Materials

- **Type status:** Other material. **Taxon:** scientificName: *Lauriacylindracea* (Da Costa, 1778); order: Stylommatophora; family: Lauriidae; **Location:** continent: Europe; islandGroup: Cyclades; island: Kea; country: Greece; countryCode: GR; **Identification:** identifiedBy: D Karakasi; **Event:** eventDate: 4/11-8/11/2021; **Record Level:** institutionCode: NHMC

##### Notes

New record from Kea.

#### Lindholmiola lens

(Férussac, 1832)

E608AFA9-D81F-5249-8781-1E52E2A50A6D

##### Materials

- **Type status:** Other material. **Taxon:** scientificName: *Lindholmiolalens* (Férussac, 1832); order: Stylommatophora; family: Helicodontidae; **Location:** continent: Europe; islandGroup: Cyclades; island: Kea; country: Greece; countryCode: GR; **Identification:** identifiedBy: D Karakasi; **Event:** eventDate: 4/11-8/11/2021; **Record Level:** institutionCode: NHMC

#### Mediterranea hydatina

(Rossmässler, 1838)

05851662-FC2F-5002-916C-AC94736E947F

##### Materials

- **Type status:** Other material. **Taxon:** scientificName: *Mediterraneahydatina* (Rossmässler, 1838); order: Stylommatophora; family: Oxychilidae; **Location:** continent: Europe; islandGroup: Cyclades; island: Kea; country: Greece; countryCode: GR; **Identification:** identifiedBy: M Mylonas; **Event:** eventDate: 4/11-8/11/2021; **Record Level:** institutionCode: NHMC

#### Monacha parumcincta

(Menke, 1828)

7B5D30C0-A8C2-592F-8A1C-D6885C3D6B02

##### Materials

- **Type status:** Other material. **Taxon:** scientificName: *Monachaparumcincta* (Menke, 1828); order: Stylommatophora; family: Hygromiidae; **Location:** continent: Europ; islandGroup: Cyclades; island: Kea; country: Greece; countryCode: GR; **Identification:** identifiedBy: K Vardinoyannis; **Event:** eventDate: 4/11-8/11/2021; **Record Level:** institutionCode: NHMC

#### Orculella critica

(Pfeiffer, 1856)

E2BB6E4E-11C0-521E-8EE5-224A9C02E136

##### Materials

- **Type status:** Other material. **Taxon:** scientificName: *Orculellacritica* (Pfeiffer, 1856); order: Stylommatophora; family: Orculidae; **Location:** continent: Europe; islandGroup: Cyclades; island: Kea; country: Greece; countryCode: GR; **Identification:** identifiedBy: L Maroulis; **Event:** eventDate: 4/11-8/11/2021; **Record Level:** institutionCode: NHMC

#### Oxychilus cyprius

(Pfeiffer, 1847)

D37988BE-8505-501E-9629-C3B4CBBAEBC8

##### Materials

- **Type status:** Other material. **Taxon:** scientificName: *Oxychiluscyprius* (Pfeiffer, 1847); order: Stylommatophora; family: Oxychilidae; **Location:** continent: Europe; islandGroup: Cyclades; island: Kea; country: Greece; countryCode: GR; **Identification:** identifiedBy: M Mylonas; **Event:** eventDate: 4/11-8/11/2021; **Record Level:** institutionCode: NHMC

#### Oxyloma elegans

(Risso, 1826)

E585ADC0-2C60-5FF3-9086-69E984B1E2E2

##### Materials

- **Type status:** Other material. **Taxon:** scientificName: *Oxylomaelegans* (Risso, 1826); order: Stylommatophora; family: Succineidae; **Location:** continent: Europe; islandGroup: Cyclades; island: Kea; country: Greece; countryCode: GR; **Identification:** identifiedBy: M Mylonas; **Event:** eventDate: 4/11-8/11/2021; **Record Level:** institutionCode: NHMC

##### Notes

New record from Kea.

#### Paralaoma servilis

(Shuttleworth, 1852)

061D716A-B050-5139-9D18-28E9474F5638

##### Materials

- **Type status:** Other material. **Taxon:** scientificName: *Paralaomaservilis* (Shuttleworth, 1852); order: Stylommatophora; family: Punctidae; **Location:** continent: Europe; islandGroup: Cyclades; island: Kea; country: Greece; countryCode: GR; **Identification:** identifiedBy: D Karakasi; **Event:** eventDate: 4/11-8/11/2021; **Record Level:** institutionCode: NHMC

##### Notes

New record from Kea.

#### Pyramidula cephalonica

(Westerlund, 1898)

6825B48F-F8C5-5487-8B85-1AEF0ED6F375

##### Materials

- **Type status:** Other material. **Taxon:** scientificName: *Pyramidulacephalonica* (Westerlund, 1898); order: Stylommatophora; family: Pyramidulidae; **Location:** continent: Europe; islandGroup: Cyclades; island: Kea; country: Greece; countryCode: GR; **Identification:** identifiedBy: M Mylonas; **Event:** eventDate: 4/11-8/11/2021; **Record Level:** institutionCode: NHMC

##### Notes

New record from Kea.

#### Pyramidula chorismenostoma

(Westerlund & Blanc, 1879)

1AC513B7-A5E0-554C-A2A5-0C6E14A38D29

##### Materials

- **Type status:** Other material. **Taxon:** scientificName: *Pyramidulachorismenostoma* (Westerlund & Blanc, 1879); order: Stylommatophora; family: Pyramidulidae; **Location:** continent: Europe; islandGroup: Cyclades; island: Kea; country: Greece; countryCode: GR; **Identification:** identifiedBy: M Mylonas; **Event:** year: 1979; **Record Level:** institutionCode: NHMC

#### Rumina saharica

Pallary, 1901

561B6286-52AC-544D-9980-0B2C3024AAE2

##### Materials

- **Type status:** Other material. **Taxon:** scientificName: *Ruminasaharica* Pallary, 1901; order: Stylommatophora; family: Achatinidae; **Location:** continent: Europe; islandGroup: Cyclades; island: Kea; country: Greece; countryCode: GR; **Identification:** identifiedBy: L Maroulis; **Event:** eventDate: 4/11-8/11/2021; **Record Level:** institutionCode: NHMC

#### Rupestrella philippii

(Cantraine, 1841)

987E0FA7-7FD5-5AA2-9B3F-F512D3DDD085

##### Materials

- **Type status:** Other material. **Taxon:** scientificName: *Rupestrellaphilippii* (Cantraine, 1841); order: Stylommatophora; family: Chondrinidae; **Location:** continent: Europe; islandGroup: Cyclades; island: Kea; country: Greece; countryCode: GR; **Identification:** identifiedBy: D Karakasi; **Event:** eventDate: 4/11-8/11/2021; **Record Level:** institutionCode: NHMC

#### Tandonia sowerbyi

(Férussac, 1823)

DA6CB85F-0290-5A7F-AFF3-B42D4AB37639

##### Materials

- **Type status:** Other material. **Taxon:** scientificName: *Tandoniasowerbyi* (Férussac, 1823); order: Stylommatophora; family: Milacidae; **Location:** continent: Europe; islandGroup: Cyclades; island: Kea; country: Greece; countryCode: GR; **Identification:** identifiedBy: K Vardinoyannis; **Event:** eventDate: 4/11-8/11/2021; **Record Level:** institutionCode: NHMC

#### Theba pisana

(Müller, 1774)

18E226B9-3A2B-5506-834E-24EC2DFF5209

##### Materials

- **Type status:** Other material. **Taxon:** scientificName: *Thebapisana* (Müller, 1774); order: Stylommatophora; family: Helicidae; **Location:** continent: Europe; islandGroup: Cyclades; island: Kea; country: Greece; countryCode: GR; **Identification:** identifiedBy: L Maroulis; **Event:** eventDate: 4/11-8/11/2021; **Record Level:** institutionCode: NHMC

##### Notes

New record from Kea.

#### Thiessea sphaeriostoma

(Bourguignat, 1857)

C50FF403-873A-5FB8-9F62-55526109D66A

##### Materials

- **Type status:** Other material. **Taxon:** scientificName: *Thiesseasphaeriostoma* (Bourguignat, 1857); order: Stylommatophora; family: Helicidae; **Location:** continent: Europe; islandGroup: Cyclades; island: Kea; country: Greece; countryCode: GR; **Identification:** identifiedBy: M Mylonas; **Event:** eventDate: 4/11-8/11/202; **Record Level:** institutionCode: NHMC

##### Notes

*Thiesseacyclolabris* mentioned by Martens (1889) should be regarded as a synonym of *T.sphaeriostoma*, after the revision of the genus by Subai (1996).

#### Trochoidea pyramidata

(Draparnaud, 1805)

B01FA4C2-915A-51FD-812B-15C14B511CE7

##### Materials

- **Type status:** Other material. **Taxon:** scientificName: *Trochoideapyramidata* (Draparnaud, 1805); order: Stylommatophora; family: Geomitridae; **Location:** continent: Europe; islandGroup: Cyclades; island: Kea; country: Greece; countryCode: GR; **Identification:** identifiedBy: M Mylonas; **Event:** eventDate: 4/11-8/11/202; **Record Level:** institutionCode: NHMC

#### Truncatellina cylindrica

(Férussac, 1807)

5E6A54B5-C043-5D65-A35C-9371AC5F6B4B

##### Materials

- **Type status:** Other material. **Taxon:** scientificName: *Truncatellinacylindrica* (Férussac, 1807); order: Stylommatophora; family: Truncatellinidae; **Location:** continent: Europe; islandGroup: Cyclades; island: Kea; country: Greece; countryCode: GR; **Identification:** identificationID: D Karakasi; **Event:** eventDate: 4/11-8/11/2021; **Record Level:** institutionCode: NHMC

##### Notes

New record from Kea.

#### Vitrea clessini

(Hesse, 1882)

D2497CCA-8789-52AB-9E46-189213E51787

##### Materials

- **Type status:** Other material. **Taxon:** scientificName: *Vitreaclessini* (Hesse, 1882); order: Stylommatophora; family: Pristilomatidae; **Location:** continent: Europe; islandGroup: Cyclades; island: Kea; country: Greece; countryCode: GR; **Identification:** identifiedBy: M Mylonas; **Event:** eventDate: 4/11-8/11/2021; **Record Level:** institutionCode: NHMC

#### Vitrea contracta

(Westerlund, 1871)

D98989CD-4105-5138-99F0-83F00F63C1E6

##### Materials

- **Type status:** Other material. **Taxon:** scientificName: *Vitreacontracta* (Westerlund, 1871); order: Stylommatophora; family: Pristilomatidae; **Location:** continent: Europe; islandGroup: Cyclades; island: Kea; country: Greece; countryCode: GR; **Identification:** identifiedBy: M Mylonas; **Event:** eventDate: 4/11-8/11/2021; **Record Level:** institutionCode: NHMC

##### Notes

New record from Kea.

#### Vitrea keaana

Riedel & Mylonas, 1981

883EE279-26C5-54FC-946B-7B39AE06E2C2

##### Materials

- **Type status:** Other material. **Taxon:** scientificName: *Vitreakeaana* Riedel & Mylonas, 1981; order: Stylommatophora; family: Pristilomatidae; **Location:** continent: Europe; islandGroup: Cyclades; island: Kea; country: Greece; countryCode: GR; decimalLatitude: 37.5597; decimalLongitude: 24.3296; **Identification:** identifiedBy: M Mylonas; **Event:** eventDate: 4/11-8/11/2021; **Record Level:** institutionCode: NHMC
- **Type status:** Other material. **Taxon:** scientificName: *Vitreakeaana* Riedel & Mylonas, 1981; order: Stylommatophora; family: Pristilomatidae; **Location:** continent: Europe; islandGroup: Cyclades; island: Kea; country: Greece; countryCode: GR; decimalLatitude: 37.5813; decimalLongitude: 24.3435; **Identification:** identifiedBy: M Mylonas; **Event:** eventDate: 4/11-8/11/2021; **Record Level:** institutionCode: NHMC

##### Notes

**Figure 2.**
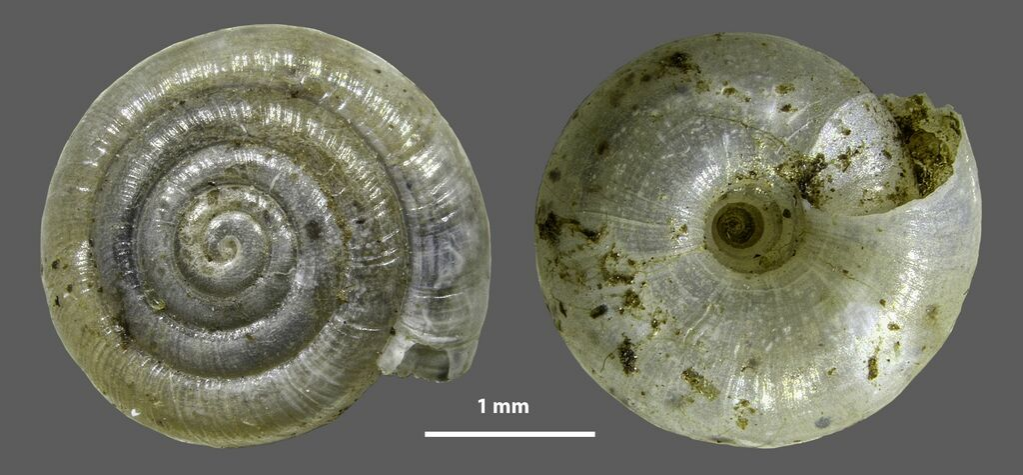
Digital microscopic images of *Vitreakeaana* Riedel & Mylonas, 1981. Specimen from Poles (37.5597, 24.3296).

Fig. 2. The only endemic land snail of Kea.

#### Vitrina pellucida

(Müller, 1774)

1304BA28-7372-5F9F-9C93-042D05D6CEAB

##### Materials

- **Type status:** Other material. **Taxon:** scientificName: *Vitrinapellucida* (Müller, 1774); order: Stylommatophora; family: Vitrinidae; **Location:** continent: Europe; islandGroup: Cyclades; island: Kea; country: Greece; countryCode: GR; **Identification:** identifiedBy: M Mylonas; **Event:** eventDate: 4/11-8/11/202; **Record Level:** institutionCode: NHMC

##### Notes

New record from Kea and the Aegean Islands.

#### Xerotricha conspurcata

(Draparnaud, 1801)

A6A321D4-A54C-52A6-9562-72FC68A1F3C3

##### Materials

- **Type status:** Other material. **Taxon:** scientificName: *Xerotrichaconspurcata* (Draparnaud, 1801); order: Stylommatophora; family: Geomitridae; **Location:** continent: Europe; islandGroup: Cyclades; island: Kea; country: Greece; countryCode: GR; **Identification:** identifiedBy: M Mylonas; **Event:** eventDate: 4/11-8/11/2021; **Record Level:** institutionCode: NHMC

##### Notes

New record from Kea.

## Analysis

### Faunistic results

Our study found 42 land snail species in Kea, belonging to 34 genera. The most species-rich genus is *Deroceras* with four species. The complete list of species is shown in the checklist section, with 11 species constituting new records for the island. Furthermore, *Deroceraspseudopanormitanum* and *Vitrinapellucida* are reported for the first time from an Aegean island.

## Discussion

Our sampling on Kea, an arguably well-surveyed island, yielded an impressive number of 11 additional land snail species, thus increasing its species richness by ~ 30% compared to that previously reported (i.e. 31 land snail species, Mylonas 1982). The recovery of 11 new species records could be mainly attributed to two different factors. First, some species known to be anthropophilous (e.g. *Xerotrichaconspurcata*, *Thebapisana*, *Oxylomaelegans*) were not previously present on the island and have probably colonised it in the period between the two samplings, potentially through human direct or indirect assistance. Second, some small-sized species – readily missed unless deliberately looked for (e.g. *Vitreacontracta*, *Paralaomaservillis*, *Truncatellinacylindrica*, *Cecilioidestumulorum*, *Lauriacylindracea*) – have been discovered thanks to the inspection of litter and soil, a practice not systematically adopted in previous sampling attempts, but necessary in order to recover the entire malacofauna of a region (Cameron and Pokryszko 2005).

Our results call attention to potential inaccurate estimations of island-level species richness due to undocumented species presences. In general, the main causes for incomplete data are sampling during unfavourable periods and inadequate sampling effort (Triantis et al. 2008). The most favourable sampling period for land gastropods in the Aegean area is from October to April, when snails are active (Mylonas 1982). Two major problems arise from sampling during unfavourable periods: first, a number of species will not be collected, since they probably aestivate hidden, either deep in the ground or in rock crevices and second, the limited number of live specimens in the samples. These shortcomings are strongly correlated to underestimation of species richness and misidentification of species, respectively (Triantis et al. 2008). Furthermore, as snails on islands can usually be located in very limited areas or habitats, recovery of full island-level species lists requires thorough examination of a complete array of available ecological space.

Our findings are also relevant with regards to accurately assessing global island diversity patterns. Recently, the first ever global inventory of island land snails (Proios et al. 2021) was based on a compilation of complete species lists from 727 islands, including 11,139 species, approximately 48% of all known land snail species. In this context, our work serves as a reminder that such attempts – as much as being essential to address the implications of large scale macroecological patterns – have their basis on rigorous local-scale faunistic studies that set the solid foundations required for large databases to be based on robust data.

## Supplementary Material

XML Treatment for Albinaria discolor

XML Treatment for Albinaria turrita

XML Treatment for Cantareus apertus

XML Treatment for Caracollina lenticula

XML Treatment for Candidula syrensis

XML Treatment for Cecilioides acicula

XML Treatment for Cecilioides tumulorum

XML Treatment for Cernuella virgata

XML Treatment for Chondrula bergeri

XML Treatment for Chondrus zebrulus

XML Treatment for Cochlicella acuta

XML Treatment for Cornu aspersum

XML Treatment for Deroceras keanense

XML Treatment for Deroceras laeve

XML Treatment for Deroceras pseudopanormitanum

XML Treatment for Deroceras seriphium

XML Treatment for Eobania vermiculata

XML Treatment for Granopupa granum

XML Treatment for Helix figulina

XML Treatment for Idyla bicristata

XML Treatment for Lauria cylindracea

XML Treatment for Lindholmiola lens

XML Treatment for Mediterranea hydatina

XML Treatment for Monacha parumcincta

XML Treatment for Orculella critica

XML Treatment for Oxychilus cyprius

XML Treatment for Oxyloma elegans

XML Treatment for Paralaoma servilis

XML Treatment for Pyramidula cephalonica

XML Treatment for Pyramidula chorismenostoma

XML Treatment for Rumina saharica

XML Treatment for Rupestrella philippii

XML Treatment for Tandonia sowerbyi

XML Treatment for Theba pisana

XML Treatment for Thiessea sphaeriostoma

XML Treatment for Trochoidea pyramidata

XML Treatment for Truncatellina cylindrica

XML Treatment for Vitrea clessini

XML Treatment for Vitrea contracta

XML Treatment for Vitrea keaana

XML Treatment for Vitrina pellucida

XML Treatment for Xerotricha conspurcata

F6307E4C-B100-5557-BBCA-EB9FB7904BAD 10.3897/BDJ.10.e87720.suppl1 Supplementary material 1 Suppl. Table 1. Collection sites Data type Sampling File: oo_714252.docx https://binary.pensoft.net/file/714252 Maroulis, Vardinoyannis, Karakasi, Proios, Mylonas, Triantis
