# Supplementary material for: The land snails (Mollusca, Gastropoda) of Kea island (Aegean, Greece)
Source: Biodivers Data J. 2022 Sep 9;10:e87720. doi: 10.3897/BDJ.10.e87720 (PMC9848511; doi:10.3897/BDJ.10.e87720)
Supplement: Supplementary material 1 — Suppl. Table 1. Collection sites [file bdj-10-e87720-s001.docx]

| **Locality** | **Coordinates** | **Main habitats** | **Species** |
| --- | --- | --- | --- |
| Platys Gialos | 37.528  24.2766 | *Shrublands with calcareous rocks and stones* | *Albinaria turrita, Candidula syrensis, Caracollina lenticula, Cecilioides acicula, Cecilioides tumulorum, Cernuella virgata, Chondrus zebrulus, Granopupa granum, Helix figulina, Lindholmiola lens, Monacha parumcincta, Orculella critica, Rumina saharica, Trochoidea pyramidata, Vitrea clessini* |
| 1.5 km SE of aerial lighthouse | 37.5483  24.2974 | *Phrygana and old cultivations, calcareous rocks and stones* | *Albinaria turrita, Cernuella virgata, Chondrula bergeri, Chondrus zebrulus, Cornu aspersum, Helix figulina, Lindholmiola lens, Monacha parumcincta, Orculella critica, Rumina saharica, Thiessea sphaeriostoma, Trochoidea pyramidata* |
| Aerial lighthouse | 37.5566  24.296 | *Phrygana, with calcareous rocks and stones* | *Albinaria turrita, Chondrula bergeri, Chondrus zebrulus, Granopupa granum, Idyla bicristata, Lindholmiola lens, Mediterranea hydatina, Monacha parumcincta, Rumina saharica, Thiessea sphaeriostoma, Trochoidea pyramidata* |
| Kampi | 37.5626  24.2838 | *Coastal area and hills around with phrygana, limestone* | *Albinaria turrita, Caracollina lenticula, Cernuella virgata, Cornu aspersum, Deroceras sp. juv., Eobania vermiculata, Granopupa granum, Lindholmiola lens, Monacha parumcincta, Orculella critica, Rumina saharica, Tandonia sowerbyi, Thiessea sphaeriostoma, Vitrea clessini, Xerotricha conspurcata* |
| Avythos | 37.5497  24.2764 | *Beach and slopes with phrygana and calcareous rocks and stones* | *Albinaria turrita, Caracollina lenticula, Cernuella virgata, Chondrula bergeri, Chondrus zebrulus, Eobania vermiculata, Granopupa granum, Helix figulina, Lindholmiola lens, Monacha parumcincta, Rumina saharica, Thiessea sphaeriostoma, Trochoidea pyramidata, Vitrea clessini* |
| Ioulis | 37.6428  24.3417 | In and all around the village | *Albinaria turrita, Cernuella virgata, Cornu aspersum, Eobania vermiculata, Idyla bicristata, Lindholmiola lens, Mediterranea hydatina, Monacha parumcincta, Orculella critica, Oxychilus cyprius, Rumina saharica, Thiessea sphaeriostoma, Xerotricha conspurcata* |
| Poles | 37.5597  24.3296 | *Archaeological site and temporary stream with phrygna, abandonded cultivations calcareous rocks* | *Albinaria turrita, Caracollina lenticula, Cecilioides tumulorum, Cernuella virgata, Cornu aspersum, Granopupa granum, Mediterranea hydatina, Idyla bicristata, Lindholmiola lens, Monacha parumcincta, Orculella critica, Paralaoma servilis, Pyramidula cephalonica, Rumina saharica, Rupestrella philippii, Thiessea sphaeriostoma, Trochoidea pyramidata, Truncatellina cylindrica, Vitrea clessini, Vitrea contracta, Vitrea keaana, Vitrina pellucida, Xerotricha conspurcata* |
| Agios Symeon, peak | 37.5813  24.3435 | *Phrygana and abandoned cultivations with calcareous rocks and stones* | *Albinaria turrita, Caracollina lenticula, Cernuella virgata, Granopupa granum, Helix figulina, Idyla bicristata, Lindholmiola lens, Mediterranea hydatina, Monacha parumcincta, Orculella critica, Rumina saharica, Rupestrella philippii, Thiessea sphaeriostoma, Trochoidea pyramidata, Vitrea clessini, Vitrea contracta, Vitrea keaana, Vitrina pellucida* |
| Profitis Ilias Mt. | 37.6207  24.3601 | *Maquis* | *Lindholmiola lens, Monacha parumcincta, Vitrea clessini* |
| Kastriani | 37.6656  24.3966 | *Phrygana, limestone rocks and stones* | *Albinaria turrita, Cornu aspersum, Deroceras pseudopanormitanum, Idyla bicristata, Lindholmiola lens, Mediterranea hydatina, Monacha parumcincta, Orculella critica, Rumina saharica, Rupestrella philippii, Thiessea sphaeriostoma, Trochoidea pyramidata, Vitrea clessini* |
| Quarry before Kastriani | 37.6744  24.3778 | *Old quarry, phrygana with calcareous rocks and stones* | *Albinaria turrita, Chondrus zebrulus, Granopupa granum, Idyla bicristata, Lindholmiola lens, Monacha parumcincta, Orculella critica, Rumina saharica, Rupestrella philippii, Thiessea sphaeriostoma* |
| Otzias yards | 37.6701  24.3497 | *Peri-urban area, home yards* | *Cantareus apertus, Caracollina lenticula, Eobania vermiculata, Xerotricha conspurcata* |
| Pisses | 37.5992  24.2776 | *Settlement,*  *abandoned cultivations,*  *shrubland on the sides* | *Albinaria turrita, Candidula syrensis, Cantareus apertus, Caracollina lenticula, Cecilioides acicula, Cecilioides tumulorum, Cernuella virgata, Chondrus zebrulus, Cochlicella acuta, Cornu aspersum, Eobania vermiculata, Granopupa granum, Idyla bicristata, Lindholmiola lens, Mediterranea hydatina, Monacha parumcincta, Orculella critica, Rumina saharica, Theba pisana, Thiessea sphaeriostoma, Trochoidea pyramidata, Vitrea clessini, Xerotricha conspurcata* |
| Agia Marina | 37.6174  24.3024 | *Around the monastery, shrubland, old cultivations and temporary stream, limestones* | *Caracollina lenticula, Cecilioides acicula, Cecilioides tumulorum, Cernuella virgata, Cornu aspersum, Deroceras keaense, Idyla bicristata, Lauria cylindracea, Lindholmiola lens, Mediterranea hydatina, Monacha parumcincta, Orculella critica, Paralaoma servilis, Rumina saharica, Tandonia sowerbyi, Thiessea sphaeriostoma, Vitrea clessini, Xerotricha conspurcata* |
| Flea, around the spring | 37.638  24.3136 | *Spring,*  *riparian vegetation* | *Caracollina lenticula, Cernuella virgata, Eobania vermiculata, Helix figulina, Lauria cylindracea, Lindholmiola lens, Mediterranea hydatina, Monacha parumcincta, Oxyloma elegans, Thiessea sphaeriostoma, Trochoidea pyramidata, Xerotricha conspurcata* |
| Vourkari | 37.6701  24.3269 | *Village and peri-urban area* | *Caracollina lenticula, Cernuella virgata, Cochlicella acuta, Deroceras seriphium, Eobania vermiculata, Idyla bicristata, Lauria cylindracea, Lindholmiola lens, Mediterranea hydatina, Monacha parumcincta, Rumina saharica, Thiessea sphaeriostoma, Trochoidea pyramidata, Vitrea clessini, Xerotricha conspurcata* |
| Korrisia meadows | 37.6565  24.313 | *Peri-urban area* | *Cantareus apertus, Caracollina lenticula, Cernuella virgata, Cochlicella acuta, Eobania vermiculata, Mediterranea hydatina, Rumina saharica, Thiessea sphaeriostoma, Xerotricha conspurcata* |
| Korrisia west slopes | 37.6616  24.3115 | *Peri-urban area, shrublands* | *Albinaria turrita, Cantareus apertus, Caracollina lenticula, Cernuella virgata, Eobania vermiculata, Lindholmiola lens, Rumina saharica, Xerotricha conspurcata* |
| Otzias coast and meadows | 37.6745  24.3491 | *Coastal area, marsh and old cultivations* | *Cantareus apertus, Caracollina lenticula, Cernuella virgata, Eobania vermiculata Lindholmiola lens, Monacha parumcincta, Orculella critica, Rumina saharica, Theba pisana, Thiessea sphaeriostoma, Trochoidea pyramidata, Vitrea clessini, Xerotricha conspurcata* |
| Korrisia east slopes | 37.6612  24.3241 | *Peri-urban area, shrublands* | *Albinaria turrita, Caracollina lenticula, Cernuella virgata, Chondrus zebrulus, Cochlicella acuta, Eobania vermiculata, Helix figulina, Lindholmiola lens, Mediterranea hydatina, Monacha parumcincta, Orculella critica, Oxychilus cyprius, Rumina saharica, Trochoidea pyramidata, Vitrea clessini, Xerotricha conspurcata* |
| Korrisia southwest slopes | 37.653  24.308 | *Peri-urban area, shrublands* | *Albinaria turrita, Cantareus apertus, Cochlicella acuta, Idyla bicristata, Lindholmiola lens, Mediterranea hydatina, Monacha parumcincta, Orculella critica, Rumina saharica, Thiessea sphaeriostoma, Xerotricha conspurcata* |
| Liparo coast | 37.5541  24.2768 | *Coastal area* | *Cernuella virgata, Eobania vermiculata, Helix figulina, Xerotricha conspurcata* |
